# Automated learning of glaucomatous visual fields from OCT images using a comprehensive, segmentation-free 3D convolutional neural network model

**DOI:** 10.1038/s41598-025-98511-0

**Published:** 2025-04-18

**Authors:** Makoto Koyama, Yuta Ueno, Yoshikazu Ito, Tetsuro Oshika, Masaki Tanito

**Affiliations:** 1 Minamikoyasu Eye Clinic, 2-8-30 Minamikoyasu, Kimitsu-shi, 299-1162 Chiba Japan; 2 Ito Eye Clinic, 3-11-2, Moriya Cyuo, Moriya, Ibaraki Japan; 3 Department of Ophthalmology, Faculty of Medicine, University of Tsukuba, Tsukuba, Ibaraki Japan; 4 Department of Ophthalmology, Shimane University Faculty of Medicine, Izumo, Shimane Japan

**Keywords:** Glaucoma, Preclinical research

## Abstract

A segmentation-free 3D Convolutional Neural Network (3DCNN) model was adopted to estimate Visual Field (VF) in glaucoma cases using Optical Coherence Tomography (OCT) images. This study, conducted at a university hospital, included 6335 participants (12,325 eyes). Two models were trained, one on the Glaucoma-Specific Training Group (GTG) and one on the Comprehensive Training Group (CTG) that included various ocular conditions without manual preselection. The CTG showed significantly better performance than the GTG in estimating VF thresholds and Mean Deviation (MD) for both Humphrey Field Analyzer (HFA) 24-2 and HFA10-2 test patterns (*p* < 0.001). Strong correlations were observed between the estimated and actual VF thresholds for HFA24-2 (Pearson’s r: 0.878) and HFA10-2 (r: 0.903), as well as MD for HFA24-2 (r: 0.911) and HFA10-2 (r: 0.944) in the CTG. The CTG demonstrated lower estimation errors than the GTG and smaller errors in severe cases. The model’s performance remained relatively stable even in advanced glaucoma cases. The model’s ability to learn from a comprehensive dataset without human annotation highlights its potential for large-scale training in the future, potentially improving glaucoma assessment and monitoring in clinical practice. Further validation in external datasets and exploration in different clinical settings are warranted.

**Supplementary Information:**

The online version contains supplementary material available at 10.1038/s41598-025-98511-0.

## Introduction

Glaucoma is a leading cause of irreversible blindness worldwide, and its early detection and treatment are crucial for preventing progressive vision loss^1,2^. In routine clinical practice, visual field (VF) testing is performed using perimetry, a method that is both time-consuming and subject to considerable variability due to its performance dependent nature^3^. The variability of patient responses and the complexity of the tests often result in challenges in accurately assessing VF loss. Optical coherence tomography (OCT), which provides noninvasive visualization of the retina and optic nerve head, has emerged as a valuable tool in the diagnosis and monitoring of glaucoma^4–18^.

Traditional two-dimensional methods for estimating VF from OCT images generally take either segmentation-based or B-scan-based approaches. Segmentation-based methods rely on manual processes for checking segmentation errors and assigning diagnostic labels^6,7,12^, while B-scan-based approaches process raw OCT scans directly without the need for segmentation^5,9–11,15^. Although both approaches have demonstrated success in VF estimation when paired OCT and VF data are available, they face certain limitations. Segmentation-based methods require time-consuming manual verification of segmentation quality, while B-scan approaches may miss important structural relationships by treating each slice independently rather than analyzing the complete three-dimensional relationships in retinal structure.

Recent advances in three-dimensional (3D) convolutional neural networks (3DCNNs) offer promising opportunities to enhance VF estimation from OCT data. Although both 2D and 3D approaches require basic image quality assessment, the segmentation-free 3D approach offers distinct advantages by processing the entire OCT volume as a unified structure. This enables the model to learn complex spatial relationships that may be lost in 2D analysis while eliminating the need for layer segmentation^4,8,18^. Theoretically, provided that reliable paired OCT-VF data is available, diagnostic labels should not be necessary for training such models. However, the feasibility and impact of training with a broader, unrestricted dataset that includes various ocular conditions remain unexplored.

This investigation is particularly relevant for real-world clinical applications, where patients may present with either isolated glaucoma or glaucoma accompanied by other ocular conditions. Models trained exclusively on carefully selected cases of pure glaucoma might face challenges when applied to patients with concurrent conditions in clinical practice. Therefore, developing models using more inclusive datasets that better reflect the full spectrum of clinical presentations could be crucial for achieving practical clinical utility.

Such an approach, if successful, could significantly reduce the manual effort required in preparing training data. This reduction in preprocessing requirements would make it more feasible to acquire large-scale datasets from various medical institutions, as the need for manual labeling and case selection would be minimized. The ability to efficiently process larger datasets could potentially lead to improved model development and clinical applicability.

In this study, we compare two training strategies: one using a dataset of selected glaucoma cases and another using a comprehensive, unrestricted dataset without manual preselection. By evaluating the VF estimation accuracy of these two approaches in patients with glaucoma, we aim to investigate how training on a broader dataset influences model performance. Our goal is to assess the practical implications of this comprehensive, segmentation-free 3DCNN approach and explore its potential impact on VF estimation in clinical settings.

## Results

### Comparison of estimation performance between the CTG and the GTG

The results of our study, as shown in Table 1, demonstrate that the Comprehensive Training Group (CTG), which was trained on a comprehensive dataset including all available cases without manual preselection or the absence of ocular conditions, achieved significantly lower MAE values than the Glaucoma-Specific Training Group (GTG), which was trained exclusively on glaucoma cases, for both Humphrey Field Analyzer (HFA) 24-2 and HFA10-2 VF thresholds and their respective Mean Deviation (MD) estimations. Statistical analysis using the two-tailed Mann‒Whitney U test with Bonferroni correction revealed a statistically significant difference (*p* < 0.001) in estimation performance between the two groups for all measures, with the CTG consistently outperforming the GTG.

**Table 1:** Comparison of VF estimation performance between the GTG and the CTG.

Testing item	GTG	CTG	*p* value
HFA24-2			
Threshold RMSE (dB)	5.39 ± 2.49	4.94 ± 2.38	< 0.001
Threshold MAE (dB)	4.18 ± 2.20	3.78 ± 2.09	< 0.001
MD MAE (dB)	2.78 ± 2.57	2.47 ± 2.45	< 0.001
HFA10-2			
Threshold RMSE (dB)	5.01 ± 2.87	4.60 ± 2.60	< 0.001
Threshold MAE (dB)	3.79 ± 2.32	3.45 ± 2.07	< 0.001
MD MAE (dB)	2.42 ± 2.34	2.18 ± 2.11	< 0.001

*GTG* Glaucoma-Specific Training Group, *CTG* Comprehensive Training Group, *HFA* Humphrey field analyzer, *RMSE* root mean squared error, *MAE* mean absolute error, *VF* visual field.

All values are mean ± standard deviation. All tests employed a two-sided Mann–Whitney U test with Bonferroni correction.

These results suggest that training the 3DCNN model on a comprehensive dataset encompassing a wide range of ocular conditions, without manual preselection, may lead to improved accuracy in estimating VF thresholds and MD values in patients with glaucoma compared to training on a glaucoma-specific dataset.

### Correlation between estimated and actual VFs in the CTG

**Fig. 1:**
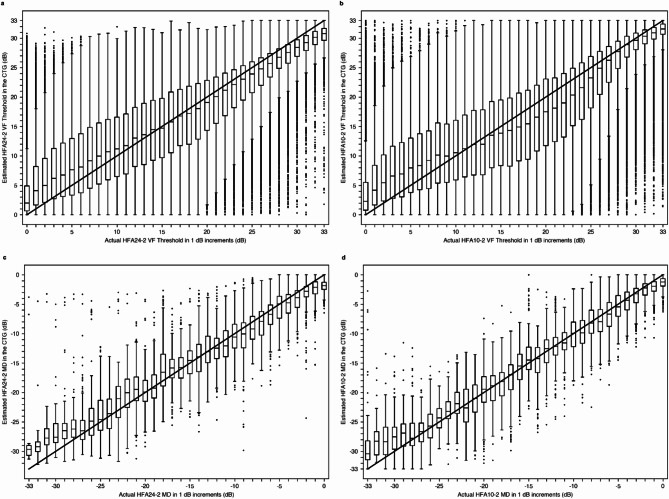
HFA VF thresholds (**a**,**b**) and MD (**c**,**d**) correlation box plots of the estimated and actual HFA VF thresholds from OCT data for HFA24-2 (**a**,**c**) and HFA10-2 (**b**,**d**) in the CTG. All the correlations were highly significant (*p* < 0.001) and positive. Spearman’s rank correlation coefficient (ρ) and Pearson’s correlation coefficient (r) were calculated for each plot. The values of (ρ, r) were (0.868, 0.878), (0.876, 0.903), (0.908, 0.911), and (0.940, 0.944) for plots (**a**), (**b**), (**c**), and (**d**), respectively. *HFA* Humphrey field analyzer, *VF* visual field, *MD* mean deviation, *OCT* optical coherence tomography, *CTG* Comprehensive training group.

Figure 1 shows the correlation between the estimated and actual VF thresholds for each test point (Fig. 1a,b) and the MD values (Fig. 1c,d) for the HFA24-2 (Fig. 1a,c) and HFA10-2 (Fig. 1b,d) data in the CTG. In all figures, the horizontal axis represents the ground truth values, while the vertical axis represents the estimated values.

The pointwise correlations for HFA24-2 and HFA10-2 yielded Pearson’s r values of 0.878 and 0.903, respectively, while the correlations for HFA24-2 MD and HFA10-2 MD yielded Pearson’s r values of 0.911 and 0.944, respectively (all *p* < 0.001). These correlations, along with their significant p-values, suggest that the CTG-trained model can estimate both pointwise VF thresholds and global VF parameters with reasonable accuracy.

### Relationship between VF severity and Estimation error in the GTG and the CTG

**Fig. 2:**
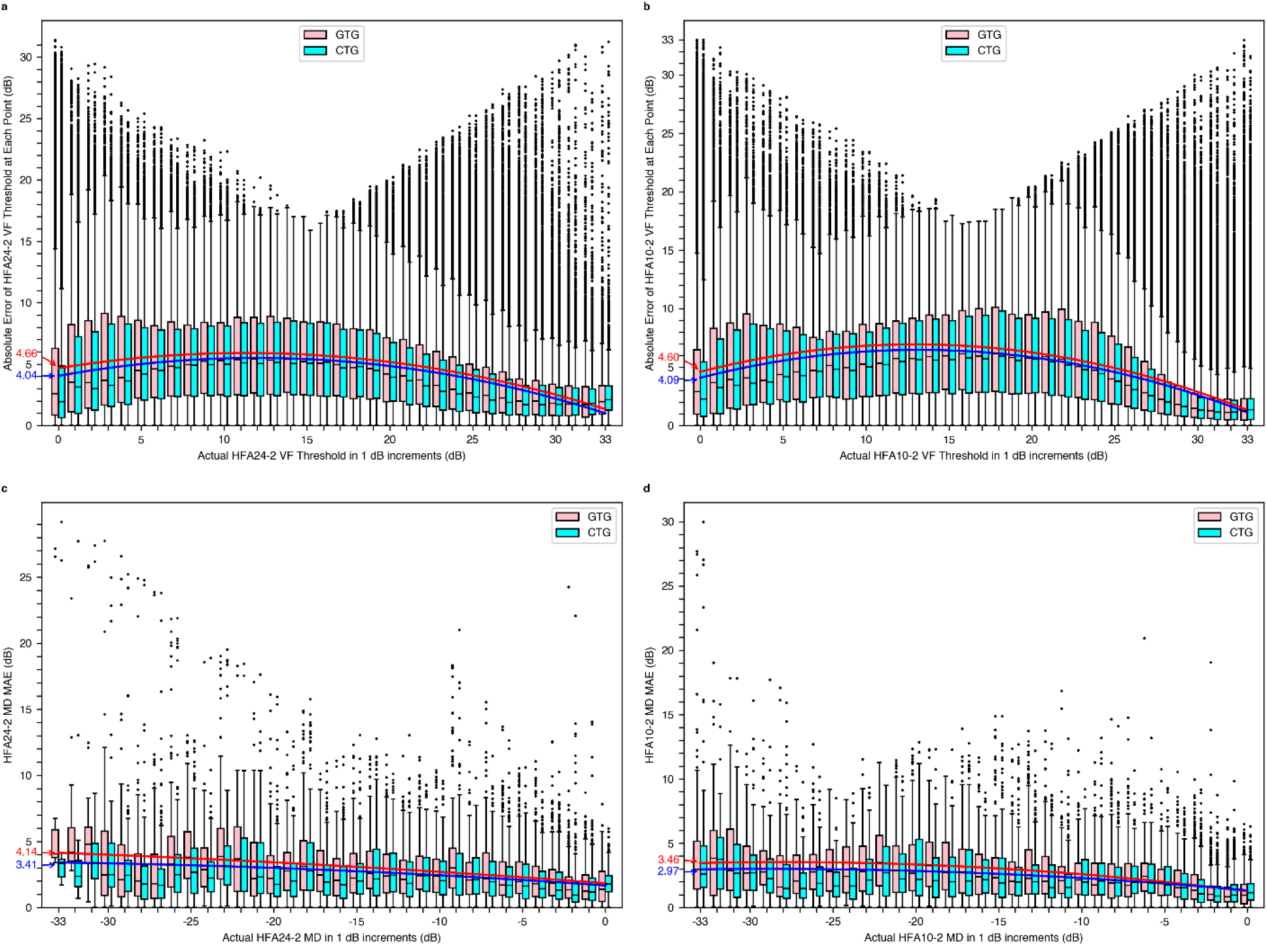
Box plots and quadratic regression lines showing the relationship between VF severity and estimation error for VF thresholds (**a**,**b**) and MD values (**c**,**d**) in the GTG and CTG for HFA24-2 (**a**,**c**) and HFA10-2 (**b**,**d**). The horizontal axis represents the actual VF thresholds or MD values, while the vertical axis represents the MAE of the estimated values. Spearman’s rank correlation coefficient (ρ) was calculated for each plot to assess the relationship between VF severity and estimation error. For HFA24-2, the ρ values were − 0.191 (GTG) and − 0.162 (CTG) for the VF thresholds and − 0.223 (GTG) and − 0.187 (CTG) for the MD values. For HFA10-2, the ρ values were − 0.360 (GTG) and − 0.333 (CTG) for the VF thresholds and − 0.347 (GTG) and − 0.252 (CTG) for the MD values. All correlations were highly significant (*p* < 0.001), indicating that estimation errors tended to increase as VF severity worsened. Consistently, the absolute value of Spearman’s ρ is smaller for the CTG than for the GTG, indicating that the estimation error tends to increase less with increasing severity. Except for a slight reversal at the MD value near 0 dB in (**d**), the lower position of the quadratic regression line for the CTG suggests that the CTG may have a smaller estimation error than the GTG. Slight upward curvatures were observed in the moderate to advanced glaucoma range, suggesting a slightly larger estimation error than in early and very advanced glaucoma cases. The difference in estimation error between the CTG and the GTG was larger in moderate to advanced glaucoma cases and smaller in early glaucoma cases. *VF* visual field, *MD* mean deviation, *GTG* Glaucoma-Specific Training Group, *CTG* Comprehensive Training Group, *HFA* Humphrey Field Analyzer, *MAE* mean absolute error.

Figure 2 shows the relationship between VF severity, indicated by the actual VF thresholds (Fig. 2a,b) and MD values (Fig. 2c,d), and estimation error, represented by the MAE of the estimated VF thresholds and MD values, for the GTG and CTG. The plots compare the performances of the GTG and CTG for HFA24-2 (Fig. 2a,c) and HFA10-2 (Fig. 2b,d).

The relationship between VF severity and estimation error, as measured by Spearman’s rank correlation coefficient (ρ), was investigated for both the GTG and the CTG. For HFA24-2, the ρ values were − 0.191 (GTG) and − 0.162 (CTG) for the VF thresholds and − 0.223 (GTG) and − 0.187 (CTG) for the MD values. Similarly, for HFA10-2, the ρ values were − 0.360 (GTG) and − 0.333 (CTG) for the VF thresholds and − 0.347 (GTG) and − 0.252 (CTG) for the MD values. All correlations were highly significant (*p* < 0.001), indicating that estimation errors tended to increase as VF severity worsened. The CTG consistently showed smaller absolute values of Spearman’s ρ compared to the GTG, indicating a lower tendency for estimation errors to increase in more severe cases.

The lower position of the quadratic regression line for the CTG indicates that the CTG consistently has smaller estimation errors than the GTG at all VF severity levels, except for the MD values around 0 dB in Fig. 2d, where there is a slight reversal. For both VF thresholds and MD values, a slight upward curvature was observed in the moderate to advanced glaucoma ranges, suggesting that estimation errors were slightly higher in these stages compared to early and very advanced cases. The difference in estimation errors between the CTG and GTG was smaller in early glaucoma cases and larger in moderate to advanced cases. Even in the most advanced glaucoma cases, the CTG maintained lower estimation errors compared to the GTG, with MAE values ranging from 2.97 to 4.09 for the CTG and 3.46 to 4.66 for the GTG at the leftmost points of the graphs.

Supplementary Fig. 2 further explores this severity-based performance by dividing the CTG data into mild (MD > − 6 dB), moderate (− 12 dB < MD ≤ − 6 dB), and severe (MD ≤ − 12 dB) subgroups. Boxplots of actual versus estimated HFA24-2 thresholds show varying correlation strengths across severity groups (mild: Pearson’s *r* = 0.711, moderate: Pearson’s *r* = 0.800, severe: Pearson’s *r* = 0.821). While the model generally performs well across all severity groups, closer examination reveals challenges in predicting localized deep defects in otherwise healthy visual fields.

### Agreement between estimated and actual MD values using Bland‒Altman plots

**Fig. 3:**
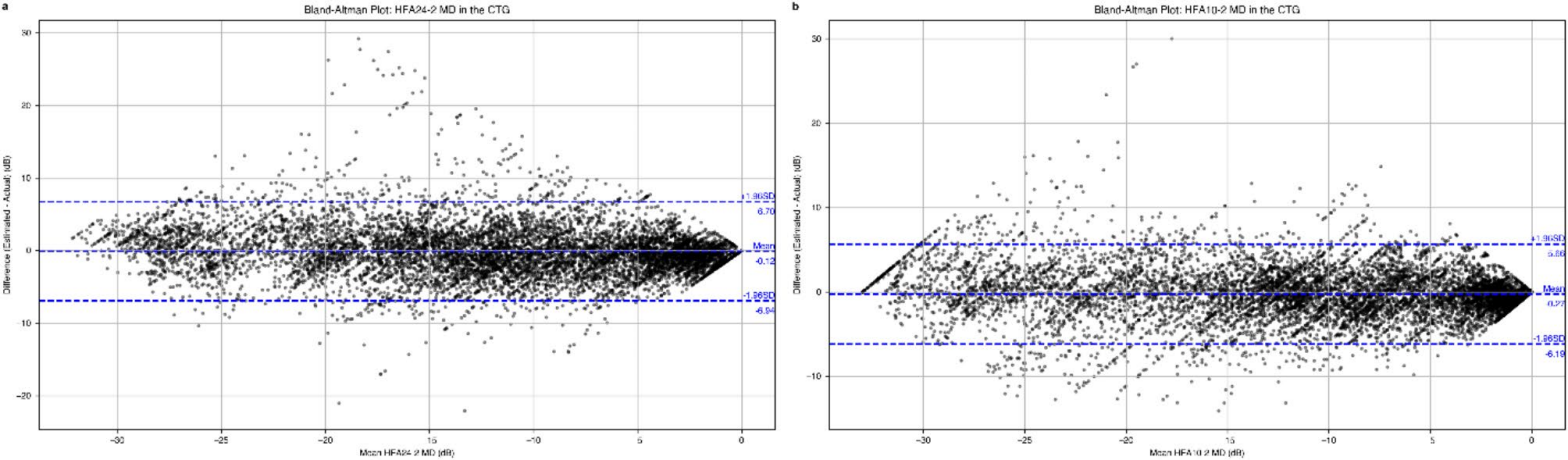
Bland‒Altman plots of MD for HFA24-2 (**a**) and HFA10-2 (**b**) in the CTG. The middle dashed line represents the mean difference (bias) between the estimated and actual MD values, while the top and bottom dashed lines represent the 95% limits of agreement (LoA). For HFA24-2, the mean difference was − 0.12 dB (95% LoA: − 6.94 to 6.70 dB). For HFA10-2, the mean difference was − 0.27 dB (95% LoA: − 6.19 to 5.66 dB). Although the data points in both plots were generally distributed around the mean difference line, a slight proportional error was observed. The correlation coefficient between the mean and difference was − 0.15 (*p* < 0.001) for HFA24-2 and − 0.12 (*p* < 0.001) for HFA10-2, indicating a small but statistically significant proportional bias, with the difference between estimated and actual MD values tending to increase slightly as the MD values decrease (i.e., becoming more negative, indicating more severe visual field loss). *MD* mean deviation, *HFA* Humphrey field analyzer, *CTG* Comprehensive Training Group.

To further assess the agreement between the estimated and actual MD values, Bland‒Altman plots were generated for both HFA24-2 and HFA10-2 (Fig. 3) in the CTG. In these plots, the differences between the estimated and actual MD values (y-axis) are plotted against their mean values (x-axis). The mean difference (bias) and the 95% limits of agreement (LoA) are also displayed.

For HFA24-2 (Fig. 3a), the Bland‒Altman plot showed a mean difference of − 0.12 dB (95% LoA: − 6.94 to 6.70 dB). Similarly, for HFA10-2 (Fig. 3b), the mean difference was − 0.27 dB (95% LoA: − 6.19 to 5.66 dB). Although the data points in both plots were generally distributed around the mean difference line, a slight proportional error was observed. The correlation coefficient between the mean and difference was − 0.15 (*p* < 0.001) for HFA24-2 and − 0.12 (*p* < 0.001) for HFA10-2, indicating a small but statistically significant proportional bias. This suggests that severe cases with lower MD values tend to have slightly larger differences between estimated and actual MD values.

### Pointwise performance and MAE heatmaps in the CTG

**Fig. 4:**
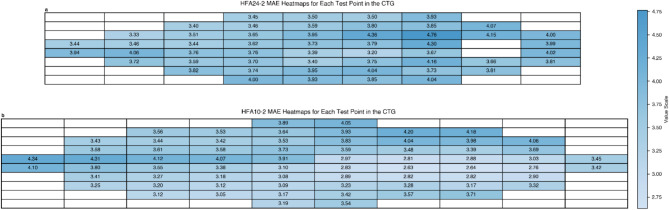
Heatmaps of the MAEs in decibels (dB) for each test point in the CTG for HFA24-2 (**a**) and HFA10-2 (**b**). The color intensity represents the magnitude of the MAE, with darker shades of blue indicating higher errors and lighter shades indicating lower errors. The corresponding MAEs are also shown for each test point. The left eye data were inverted to display as the right eye data. For HFA24-2, the test point superonasal to the blind spot had a slightly higher MAE of 4.76 dB than other locations. The HFA24-2 MAEs temporal to the blind spot and in the upper lid region were not particularly high compared to those at the other test locations. *MAE* mean absolute error, *CTG* Comprehensive Training Group, *HFA* Humphrey field analyzer.

Figure 4 shows the MAE heatmaps in decibels (dB) for each test point measured by the CTG. The left eye data were horizontally inverted and are shown as right eye data. In HFA24-2 (Fig. 4a), the MAE at the test point superonasal to the blind spot was slightly greater (4.76 dB), whereas the blind spot temporal and upper eyelid regions did not show notably high errors.

Additionally, Supplementary Fig. 4 offers pointwise boxplots comparing actual versus estimated HFA24-2 thresholds for all 52 test locations (excluding the Marriott blind spot), colored by anatomical clusters. These boxplots reveal a pattern consistent with the nerve fiber layer anatomy: nasal visual field points (colored in red for the superonasal cluster and blue for the inferonasal cluster), which correspond to temporal retinal areas well-covered by the OCT scan, show better estimation performance even in severe cases. In contrast, estimation errors tend to increase at the more peripheral temporal field points in advanced cases.

## Discussion

In our study, the CTG, which was trained on a comprehensive dataset including various ocular conditions without manual preselection, showed better estimation performance than the GTG, which was trained only on glaucoma cases, for both HFA24-2 and HFA10-2 VF thresholds and their respective MD values. As far as we could find, no previous studies have reported the use of a comprehensive dataset such as the CTG to train a 3DCNN model for VF estimation from OCT images. This novel aspect of our study highlights the potential benefits of using diverse training data to develop more robust and generalizable deep learning models for glaucoma assessment. These results suggest that the inclusion of different ocular conditions in the training dataset, without the need for manual preselection, may contribute to the ability of the segmentation-free 3DCNN model to estimate VF in patients with glaucoma with reasonable accuracy, potentially improving model performance and generalizability.

Previous approaches to VF estimation from OCT have primarily followed three methodological paths: using circumpapillary OCT B-scans^5,9–11,13–15^, utilizing segmented macular thickness maps^6,7,12^, or employing wide-field OCT scans^16,17^. Each approach has shown distinct advantages and limitations in capturing structure-function relationships. Studies using circumpapillary OCT B-scans have demonstrated success in estimating HFA24-2 parameters. Mariottoni et al. achieved a mean absolute error of 4.25 dB using a 1D CNN model on B-scan images^14^, while Lazaridis et al. and Hemelings et al. showed the effectiveness of 2D CNN approaches using unsegmented circumpapillary scans^5,10^. These studies demonstrated that direct analysis of OCT B-scans could effectively capture the relationship between retinal structure and peripheral VF defects.

Research utilizing macular OCT has shown particular strength in estimating central VF parameters. Hashimoto et al., using a 9 mm × 9 mm macular scan area similar to that used in our study, demonstrated the value of combining structural information with existing HFA24-2/30 − 2 data to improve HFA10-2 predictions^12^. Our finding that the CTG model performs well in estimating central and near-peripheral field defects (except for temporal peripheral points in severe cases) builds upon these works, suggesting that the 9 mm × 9 mm scan area captures sufficient structural information for most clinically relevant test points.

Studies employing wide-field OCT scans, such as those by Moon et al. and Park et al., have demonstrated promising results in estimating both central and peripheral VFs by combining macular and peripapillary information^16,17^. This broader structural coverage can capture a wider range of nerve fiber trajectories. In contrast, our approach focuses on macular OCT scans. Future investigations might explore the potential benefits of employing 3D models with a more extensive scanning area—encompassing both the macular and peripapillary regions—to see whether such an approach could further improve VF estimation.

Our approach utilizing raw OCT volume data without segmentation builds upon several pioneering studies in 3DCNN applications. Chen et al. demonstrated the advantages of unsegmented OCT volumes, particularly in cases affected by the floor effect^8^, while Mohammadzadeh et al. and Yu et al. also demonstrated the potential of 3DCNN models in analyzing volume scans^4,18^. Our study extends these findings by demonstrating that training on a comprehensive, unselected dataset can further enhance the model’s ability to handle various disease severities. This suggests that the combination of 3D volumetric analysis with diverse training data may be particularly effective in capturing the complex structural patterns associated with glaucomatous damage.

Interestingly, our CTG model maintained relatively stable performance even in severe cases, where VF estimation has traditionally been considered more challenging. This improvement may be attributed to several factors. First, the model’s exposure to diverse pathological patterns during training may enhance its ability to recognize subtle structural variations that persist even in advanced cases. Second, the use of 3D volumetric data allows the model to learn complex spatial relationships that might be lost in segmentation-based approaches. Third, the inclusion of various ocular conditions in the training data may help the model better distinguish glaucomatous changes from other pathological alterations.

The use of 9 mm × 9 mm macular OCT scans in our study provides coverage that approximates the distance between the macula and optic disc in most eyes, enabling visualization of nerve fiber trajectories for VF points in the HFA24-2 pattern, despite individual variations in ocular anatomy. This scan area offers broader coverage than the conventional 6 mm × 6 mm protocols used in many previous studies. The spatial distribution of estimation errors (Fig. 4 and Supplementary Fig. 4) reveals a pattern that corresponds to the anatomical relationship between retinal nerve fiber layer (RNFL) trajectories and visual field points. Specifically, our analysis of pointwise estimation accuracy (Supplementary Fig. 4) suggests that the model’s performance correlates with the length of visible nerve fiber trajectories captured within the OCT scan. Test points nasal to the blind spot, which correspond to temporal retinal areas with longer visible RNFL trajectories within the scan area, demonstrated more stable performance even in severe cases. In contrast, for points temporal to the blind spot, which correspond to nasal retinal areas with shorter or partially absent RNFL trajectories in the scan, estimation errors increased significantly in cases with advanced VF loss. This spatial pattern of performance directly reflects the anatomical coverage limitations of our macular OCT scans: while the scan area thoroughly captures the nerve fiber layer trajectories corresponding to the nasal VF, the structural information for the temporal VF is incomplete as these fibers originate from retinal areas that lie beyond the nasal boundary of our OCT scan coverage. Notably, the model’s ability to estimate VF values even in areas without complete OCT coverage of their corresponding RNFL trajectories, albeit with increased errors in severe cases, suggests that it may have learned to recognize patterns of glaucomatous damage and make inferences about structural-functional relationships from the large number of structure-function pairs in the training data.

While our model shows strong overall correlations between estimated and actual VF measurements, individual predictions exhibit notable variability that requires careful consideration. As illustrated in Supplementary Fig. 2, the model demonstrates varying performance across different glaucoma severity groups. Particularly in patients with mild glaucoma (MD > − 6 dB), we observed reduced accuracy in predicting localized deep defects. The distribution analysis (Supplementary Fig. 2d) reveals that these localized severe sensitivity decreases in otherwise mild cases are relatively uncommon in our dataset, potentially contributing to the model’s difficulty in accurately predicting them. This suggests that the model may rely more heavily on global loss patterns to estimate threshold values, particularly when encountering atypical presentations of focal defects in eyes with otherwise preserved visual function. This prediction variability may arise from multiple factors including inherent VF testing inconsistencies (e.g., fixation losses, false responses), deviations from typical structure-function relationships, image quality differences, anatomical variations, and underrepresentation of certain defect patterns in the training data. The relative scarcity of localized deep defects in eyes with mild overall damage presents a particular challenge for the learning algorithm, as the model has fewer examples to learn these specific patterns. This limitation is clinically significant, as early glaucoma often manifests as localized defects before progressing to more diffuse loss patterns. Improving the model’s ability to accurately predict focal defects in otherwise healthy visual fields represents an important direction for future refinement and would enhance the clinical utility of this approach for early detection and monitoring.

While the theoretical possibility of training models using paired VF-OCT data without diagnostic labels has been recognized, most previous studies have utilized selected datasets with known diagnoses. This approach ensures that the study population is well-defined but can be resource-intensive and may introduce selection bias. Our findings suggest that training with more diverse or less strictly curated datasets could enhance model performance. This finding has significant implications for the scalability of deep learning applications in ophthalmology, as it suggests that models can be effectively trained using real-world clinical data without the need for extensive preprocessing or diagnostic verification. This could facilitate multi-center collaborations and the development of more robust models trained on increasingly diverse datasets.

However, it is important to acknowledge the limitations of our study. While our dataset is large and diverse, the single-center nature of our study may limit generalizability. Additionally, our study design cannot definitively determine whether the improved performance of the CTG project stems from increased data volume or greater condition diversity. Future multi-center studies should systematically evaluate these factors. The automatic exclusion method for upper eyelid artifacts, while providing a systematic approach to quality control, may potentially exclude valid glaucomatous superior VF defects. Further research is needed to optimize these criteria for better discrimination between artifacts and true glaucomatous defects.

In conclusion, our study suggests that a segmentation-free 3DCNN model trained on a comprehensive dataset can estimate VF in patients with glaucoma with reasonable accuracy, showing better performance than models trained exclusively on glaucoma cases. The correlations between the estimated and actual VF parameters support the model’s potential for clinical application in glaucoma assessment and monitoring. By streamlining the process of VF estimation from OCT images, our approach could help optimize patient care and resource allocation in glaucoma management. The model’s ability to learn from a comprehensive dataset without human annotation highlights its potential for large-scale training, which could lead to further improvements in performance and generalizability. This feature suggests that our approach may be particularly valuable for future research and clinical implementation, as it could reduce the need for manual data preparation and enable the inclusion of a wide range of ocular conditions. Further research is needed to validate our findings in external datasets and refine the methods for more accurate VF estimation.

## Methods

### Study design

This retrospective study was approved by the Institutional Review Board of Shimane University Hospital (IRB No. KS20230719-3, approved on August 10, 2023) and was conducted in accordance with the Declaration of Helsinki. It included all cases from October 1, 2006, to October 19, 2023, at this tertiary care center known for its specialization in glaucoma. Due to the retrospective nature of the study, the Institutional Review Board of Shimane University Hospital (IRB No. KS20230719-3) waived the need for obtaining informed consent. An opt-out option was provided, and study information was posted on the hospital’s website and premises. This study aimed to evaluate the efficacy of a segmentation-free 3DCNN model for estimating VF in patients with glaucoma using a comprehensive dataset from a high-volume university hospital.

### Participants

This study retrospectively enrolled all subjects who underwent macular map with 9 mm × 9 mm OCT and who completed VF testing within the study period. The OCT devices used included at least one of the following Nidek models: RS-3000, RS-3000 Advance, or RS-3000 Advance2. VF testing was performed using the Carl Zeiss Meditec HFA, with testing including at least one of the SITA standard protocols 30-2, 24-2, or 10-2. Following an opt-out exclusion process in which no individuals were excluded, the study initially collected data from 6335 participants representing 12,325 eyes.

### Study groups and inclusion criteria

For training purposes, participants were divided into two groups: the GTG and the CTG. Both groups were trained using data from patients with an OCT signal strength index (SSI) of 7 or higher as an initial quality criterion.

The GTG was trained exclusively on data from patients diagnosed with glaucoma. For this group, in addition to the SSI threshold, all OCT images were manually reviewed to exclude those with significant artifacts or poor image quality. The diagnosis was made on the basis of characteristic findings, including optic disc cupping and neuroretinal rim thinning on fundus photography, thinning of the retinal nerve fiber layer and ganglion cell complex on OCT imaging, and the presence of visual field defects on perimetry consistent with the fundus photography and OCT findings. Only eyes that satisfied the Anderson-Patella criteria were included in the GTG. These criteria require that there are at least three contiguous points where the pattern deviation probability is less than 5%, with at least one of these points having a probability of less than 1%, the pattern standard deviation must be less than 5%, or the results of the glaucoma hemifield test must indicate values outside normal limits^19^. Cases were restricted to those with open-angle glaucoma, including those with exfoliation glaucoma, while cases of angle-closure glaucoma, uveitic glaucoma, or eyes with acute glaucoma attacks were excluded. This restriction was implemented because angle-closure glaucoma, particularly in acute attacks, often presents discordance between OCT findings and VF results, and obtaining sufficient samples for reliable model training is challenging. Patients with other retinal diseases or upper eyelid artifacts were excluded.

The CTG was trained on a dataset that included all available cases from the university hospital, with no manual screening or exclusions based on the presence or absence of specific ocular conditions. Owing to the retrospective nature of this large-scale hospital-wide study and practical limitations, we did not individually document the specific types or combinations of ocular conditions present in each case. This approach reflects real-world clinical settings where patients may present with various ocular conditions. However, to address the potential problem of upper eyelid artifacts in the learning process, we implemented an automatic exclusion method for the CTG: we calculated the mean values of the three nasal points for each row in the HFA24-2 visual fields and then subtracted these values from those of the row directly above. If the resulting differences for each row exceeded 6 dB, 6 dB, and 8 dB from top to bottom, respectively, the visual field was considered to contain an upper eyelid artifact and was excluded from the training data. This novel approach, which was not found in previous reports, was used to ensure the quality of the training data in the CTG. The automatic exclusion of cases with upper eyelid artifacts accounted for 18.6% of the total OCT scans in the CTG. While this exclusion rate is relatively high and may potentially exclude some valid glaucomatous superior VF defects, this conservative threshold was chosen to prevent the model from learning upper eyelid artifacts during training. We acknowledge that the criteria for automatic exclusion requires further optimization to achieve a better balance between avoiding artifact-based learning and retaining valid glaucomatous defects. Given the large scale of this dataset, manual review of image quality was not feasible, and quality control was limited to the SSI threshold and an automatic exclusion method for upper eyelid artifacts. Importantly, this automatic exclusion was only applied during the training phase of the CTG model and had no effect on the evaluation dataset.

For evaluation (testing) purposes, both the GTG and the CTG were evaluated under identical conditions, focusing exclusively on patients diagnosed with glaucoma who met the same inclusion criteria as the GTG. Specifically, the evaluation was limited to glaucoma cases with an SSI ≥ 7. This approach allowed a direct comparison of the efficacy and accuracy of each training approach under uniform test conditions, assessing the impact of training on a glaucoma-specific dataset versus a comprehensive dataset that included various ocular conditions.

### Data preparation and regression fitting for accurate VF e7stimation

To create a unified dataset for training the 3DCNN, we combined the HFA24-2 and HFA10-2 data into a single pair of VF and OCT data. The peripheral areas of the HFA30-2 VFs were trimmed to match the HFA24-2 specifications. While recent studies have typically used a 15% threshold for the false-positive rate of SITA tests^4,11^, our preliminary analyses—examining not only MD but also pointwise thresholds for both HFA24-2 and HFA10-2—showed optimal estimation performance when including HFA tests with false-positive rate, false-negative rate, and fixation loss rates below 33%. Following previous works^7,15^, we applied these reliability criteria to both HFA24-2 and HFA10-2 tests and excluded tests with rates greater than or equal to 33%. To reduce VF variability, time-based regression lines for each test point were constructed when two or more VF data points were available. For cases with only a single VF test, if there was an OCT scan within 6 months before or after the VF test date, the VF values were directly paired with the corresponding OCT data and included in the dataset. Critically, to negate the learning effect—where improved test results may reflect familiarity rather than true recovery—we set positive regression slopes to zero, recognizing the irreversible nature of glaucomatous VF loss. Using these regression lines, VF thresholds were calculated corresponding to the date the OCT was taken, with visual field thresholds ranging from 0 to 33 dB and MD values ranging from 0 to − 33 dB. Because extending the regression line created from a small number of VF data would increase the error, the “validity period” was calculated as 6 months × number of tests (n), and data pairs were excluded if the interval between the OCT and the most recent visual field test was longer than the validity period. In cases where data points were missing from either HFA24-2 or HFA10-2, we assigned a mask value of 1 to indicate the absence of data. To create a unified dataset for training the 3DCNN, we combined the HFA24-2 and HFA10-2 data into a single pair of VF and OCT data, resulting in a total of 122 output points: 52 points from HFA24-2, 68 points from HFA10-2, and their respective MDs.

**Fig. 5:**
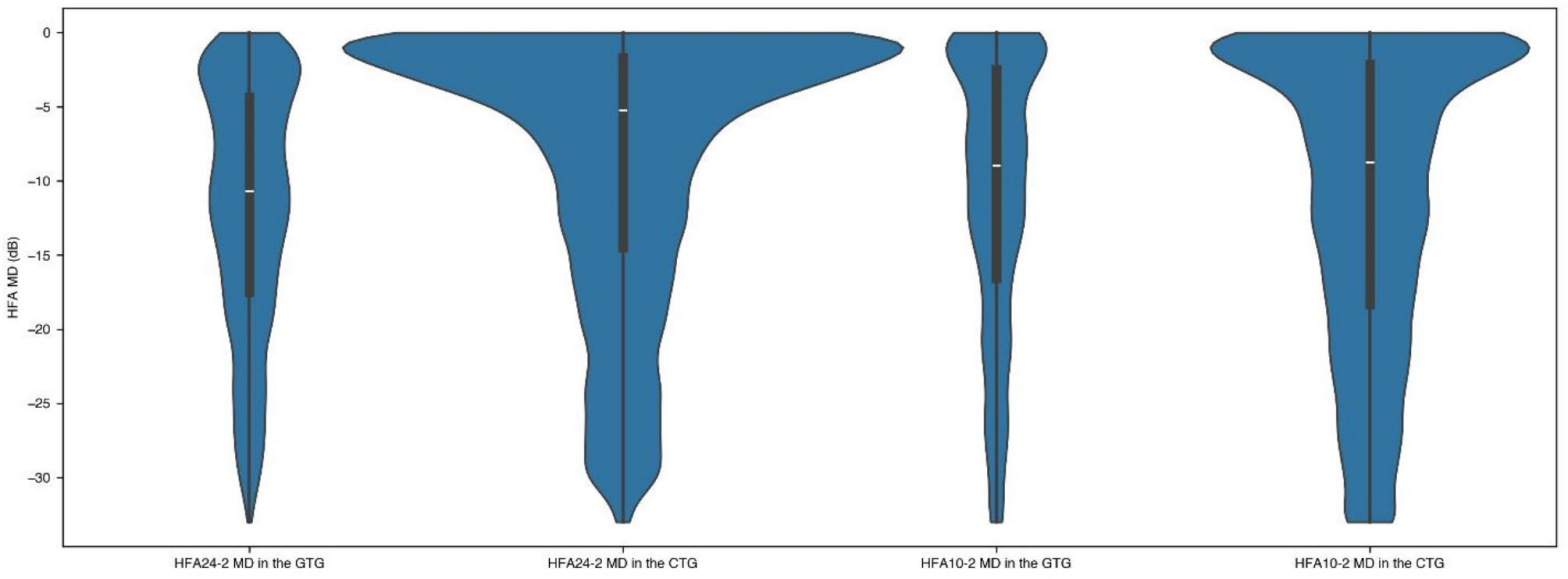
Violin plots showing the distribution of MD values for HFA24-2 and HFA10-2 in the GTG and CTG. The plots display the probability density of the MD values, with wider sections indicating a greater probability of observing that particular MD value. The horizontal axis represents the different groups and test types: HFA24-2 MD in the GTG, HFA24-2 MD in the CTG, HFA10-2 MD in the GTG, and HFA10-2 MD in the CTG. The vertical axis represents the HFA MD values in decibels (dB). *MD* mean deviation, *GTG* Glaucoma-Specific Training Group, *CTG* Comprehensive Training Group.

These data preparation and regression adjustment steps were implemented to optimize the dataset for training the 3DCNN, aiming to improve the accuracy of estimating VF from OCT images, taking into account the challenges posed by VF variability and the progressive nature of glaucoma. The demographic and clinical characteristics of the two groups are presented in Table 2. Figure 5 illustrates the distribution of MD values for HFA24-2 and HFA10-2 in the GTG and CTG, providing a visual comparison of the probability densities within each group.

**Table 2:** Patient characteristics and data distribution during model training.

Patient characteristics	GTG	CTG
Total number of sets of paired data	9771	53,698
Number of sets of paired data in HFA24-2	9414	51,911
Number of sets of paired data in HFA10-2	9247	29,522
Total number of patients	606	5490
Number of patients in HFA24-2	588	5376
Number of patients in HFA10-2	505	2211
Total number of eyes	1079	9783
Number of eyes in HFA24-2	1031	9479
Number of eyes in HFA10-2	970	3974
Mean deviation of the HFA24-2, mean ± SD (dB)	− 11.7 ± 8.42	− 8.90 ± 8.99
Mean deviation of the HFA10-2, mean ± SD (dB)	− 10.7 ± 9.16	− 11.1 ± 9.81
SSI, mean ± SD	8.69 ± 1.05	8.57 ± 1.07
Focus, mean ± SD (D)	− 2.47 ± 3.37	− 1.68 ± 3.08
Age, mean ± SD (years)	68.3 ± 12.4	69.1 ± 14.7
Number of HFA24-2 tests per eye, mean ± SD	9.14 ± 7.87	5.72 ± 6.48
Number of HFA10-2 tests per eye, mean ± SD	6.64 ± 5.00	5.19 ± 4.70
Interval between HFA24-2 tests, mean ± SD (days)	273 ± 278	253 ± 360
Interval between HFA10-2 tests, mean ± SD (days)	226 ± 164	223 ± 238
Number of OCT tests per eye, mean ± SD	9.06 ± 5.58	5.49 ± 5.54

*GTG* Glaucoma-Specific Training Group, *CTG* Comprehensive Training Group, *HFA* Humphrey field analyzer, *VF* visual field, *SD* standard deviation, *SSI* signal strength index of OCT image, *OCT* optical coherence tomography.

### 3DCNN model architecture and training strategy

We employed a 10-fold cross-validation method for our 3DCNN, ensuring a clear separation of patients randomly into training, validation, and test sets at an 8:1:1 ratio. This patient-wise separation ensured that data from the same patient did not appear in more than one set, which is critical for unbiased model evaluation. The epoch that showed optimal validation performance was selected for the final test set to leverage the model’s peak estimation ability.

**Fig. 6:**
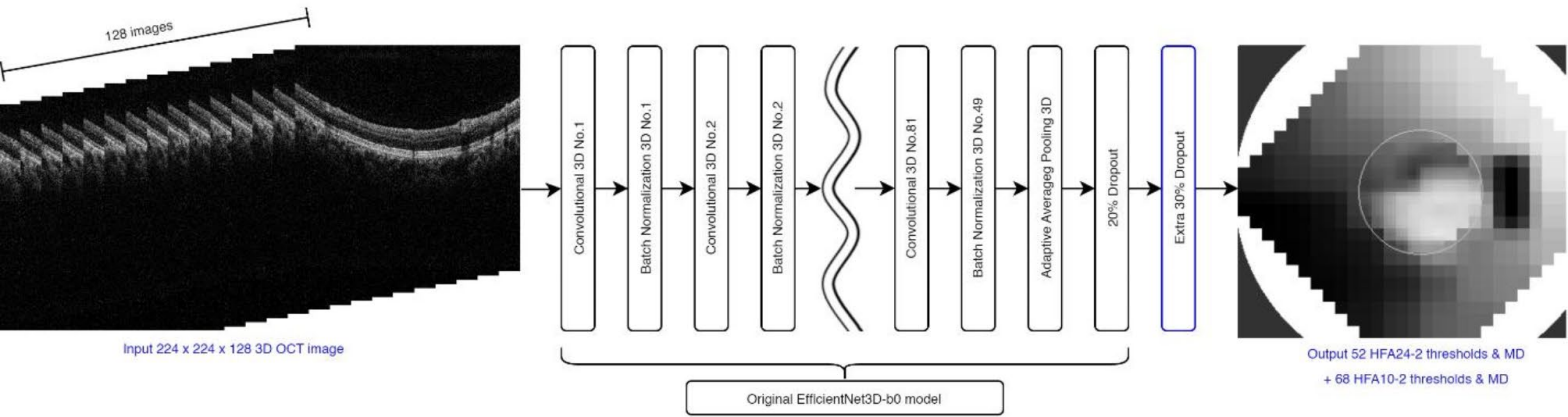
Schematic representation of the segmentation-free 3DCNN model for estimating VF from OCT images. The input 224 × 224 × 128 3D OCT image is processed through a series of 3D convolutional and batch normalization layers based on the EfficientNet3D-b0 architecture, followed by adaptive average pooling, dropout layers, and an additional 30% dropout layer to mitigate overfitting. Without any hidden layers between the final 30% dropout layer and the output, the model directly outputs 52 and 68 estimated VF thresholds along with their respective MD values for HFA24-2 and HFA10-2, allowing for end-to-end estimation of VF from 3D OCT images without manual segmentation. *3DCNN* 3D convolutional neural network, *VF* visual field, *OCT* optical coherence tomography, *MD* mean deviation.

OCT images were standardized to a resolution of 224 × 224 × 128 and normalized within a range of − 1 to 1 using min–max normalization. For the HFA24-2 and HFA10-2 datasets, z-score normalization was applied using the mean and standard deviation of the training dataset to ensure consistent data scaling across datasets. Our model is based on the EfficientNet3D-b0 architecture^20^ with an additional 30% dropout layer in the head to mitigate overfitting (Fig. 6). The model was trained from scratch with randomly initialized weights, without employing transfer learning or fine-tuning techniques. The architecture is designed to estimate a comprehensive set of 122 points: 52 from HFA24-2, 68 from HFA10-2, and their respective MDs. This approach effectively translates OCT images directly into detailed VF estimates without utilizing hidden layers.

To further diversify the dataset, we applied data augmentation techniques, including horizontal flipping of the left eye images for anatomical consistency with the right eye images. Vertical flipping was applied during training, as reported in another study^12^. Additionally, vertical flipping was consistently applied across all the data phases, including validation and testing. For the test data, the estimation accuracy was improved by averaging the results from the original and vertically flipped inputs.

### Loss function and training details

The training objective was to minimize the mean square error (MSE) between the estimated and actual VF data. To eliminate the effect of missing data, the backpropagation calculation was multiplied by (1-mask) during training, and missing data were also excluded during evaluation. This strategy ensured that model learning was unaffected by gaps in VF data, providing a framework for training under conditions of data incompleteness. The Adam optimizer was used with a mini-batch size of 4, and the learning rate was incrementally increased from 6e−4 to 1e−3 over three epochs and then decreased to 6e−4 over five epochs. The environment used was Ubuntu 22.04.2 LTS, CUDA 11.8, Python 3.11.2, and PyTorch 2.01.

### Statistical analysis

For the evaluation (testing) phase, both the GTG and the CTG were evaluated under the same conditions, using all available paired data for each eye that met the GTG inclusion criteria, even if there were multiple data pairs per eye. This approach allowed a comprehensive analysis of the model’s performance across all relevant data points, ensuring an unbiased evaluation of the estimation accuracy in glaucoma cases. We evaluated the performance of our model using the root mean squared error (RMSE), mean absolute error (MAE), Pearson’s correlation coefficient (r), and Spearman’s rank correlation coefficient (ρ). These metrics were calculated for VF thresholds and MD values to assess the accuracy and correlation between the estimated and actual values. To align with previous reports^6,7^, we calculated the RMSE for each pair of estimated and actual values before taking the average, providing a comprehensive assessment of the model’s performance on a pairwise basis. NumPy 1.25.1 and SciPy 1.10.1 were used to perform these calculations.

## Electronic supplementary material

Below is the link to the electronic supplementary material.

Supplementary Material 1

Supplementary Material 2

Supplementary Material 3

Supplementary Material 4

Supplementary Material 5

## Data Availability

The datasets generated and/or analyzed during the current study are not publicly available due to privacy and ethical considerations related to patient data, as the study was conducted on an opt-out basis without obtaining explicit consent from all participants for the release of their raw data, but aggregate or anonymized data are available from the corresponding author on reasonable request.
