# Supplementary figures and images for: Automated learning of glaucomatous visual fields from OCT images using a comprehensive, segmentation-free 3D convolutional neural network model
Source: Sci Rep. 2025 Apr 18;15:13395. doi: 10.1038/s41598-025-98511-0 (PMC12008402; doi:10.1038/s41598-025-98511-0)

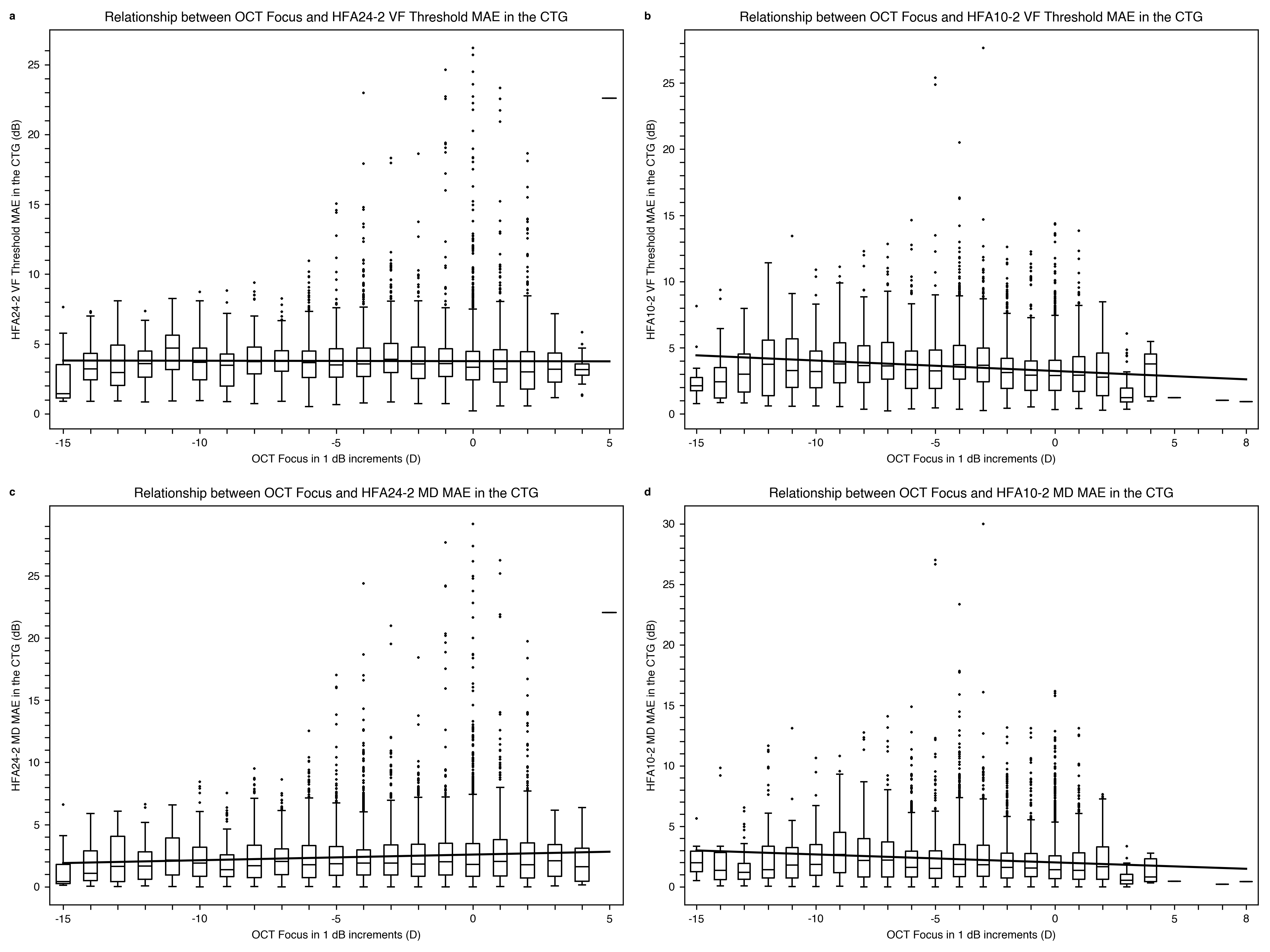

Supplement: Supplementary file 1 — Supplementary Material 1 [file 41598_2025_98511_MOESM1_ESM.tiff]

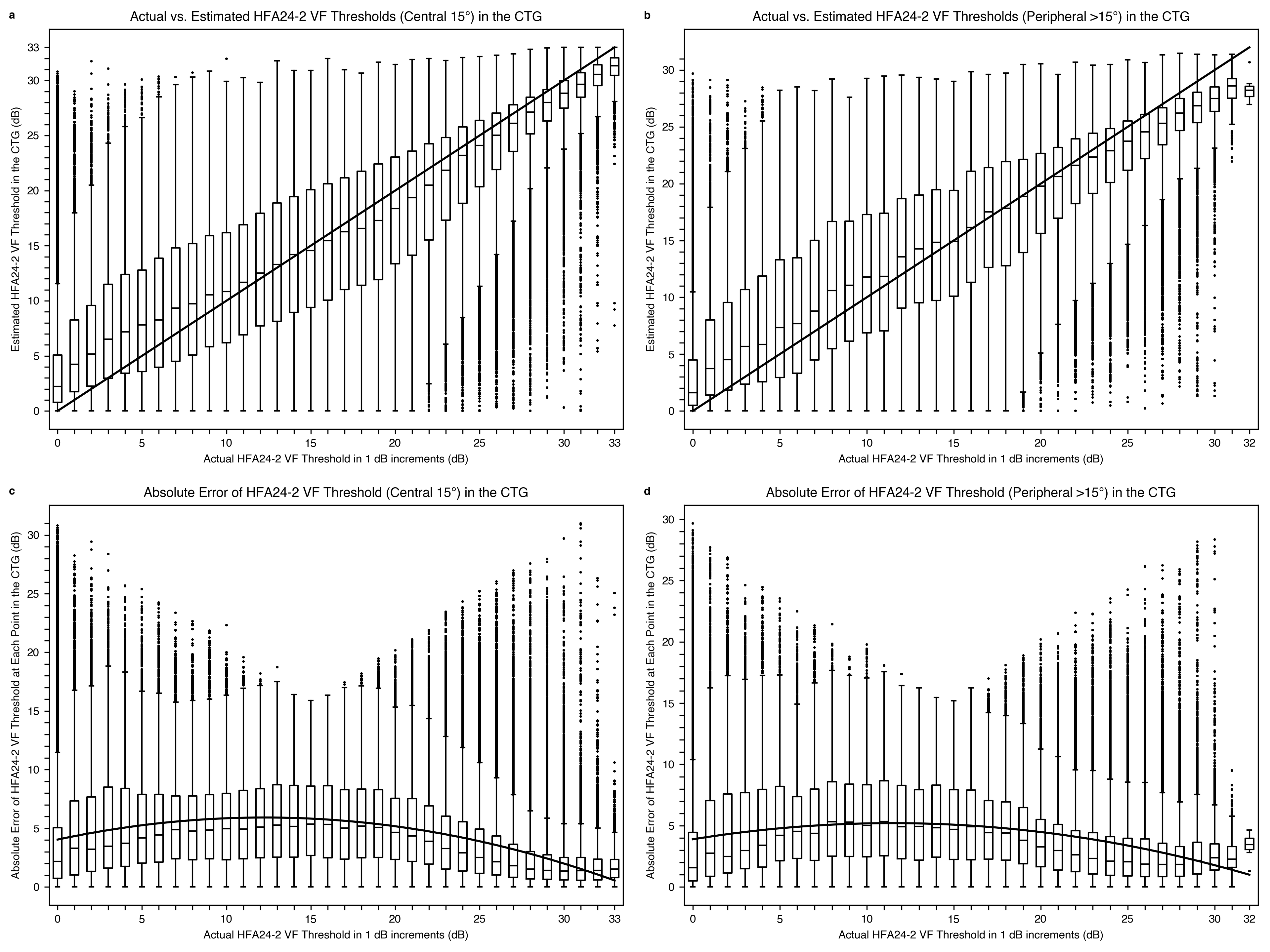

Supplement: Supplementary file 2 — Supplementary Material 2 [file 41598_2025_98511_MOESM2_ESM.tiff]

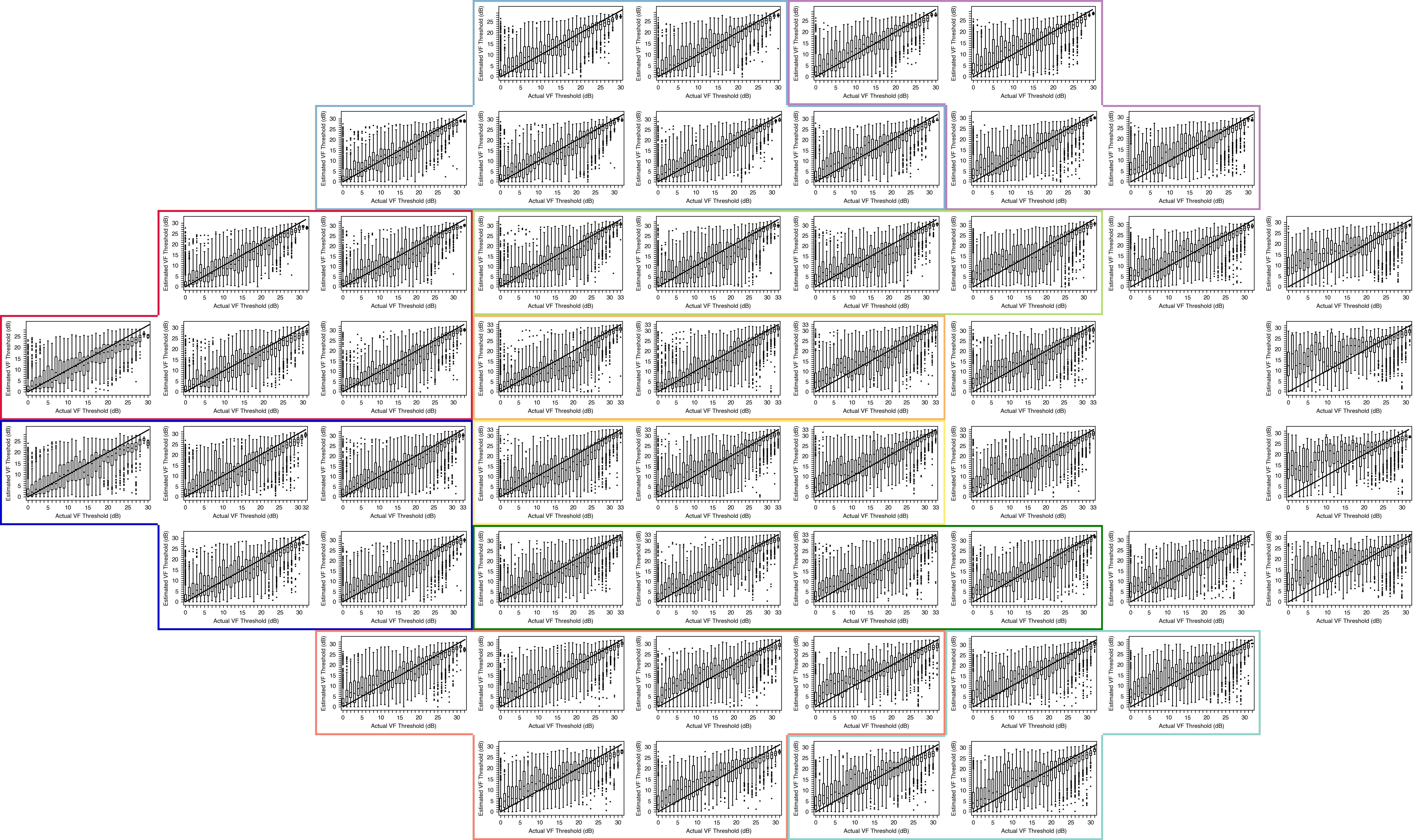

Supplement: Supplementary file 3 — Supplementary Material 3 [file 41598_2025_98511_MOESM3_ESM.tiff]

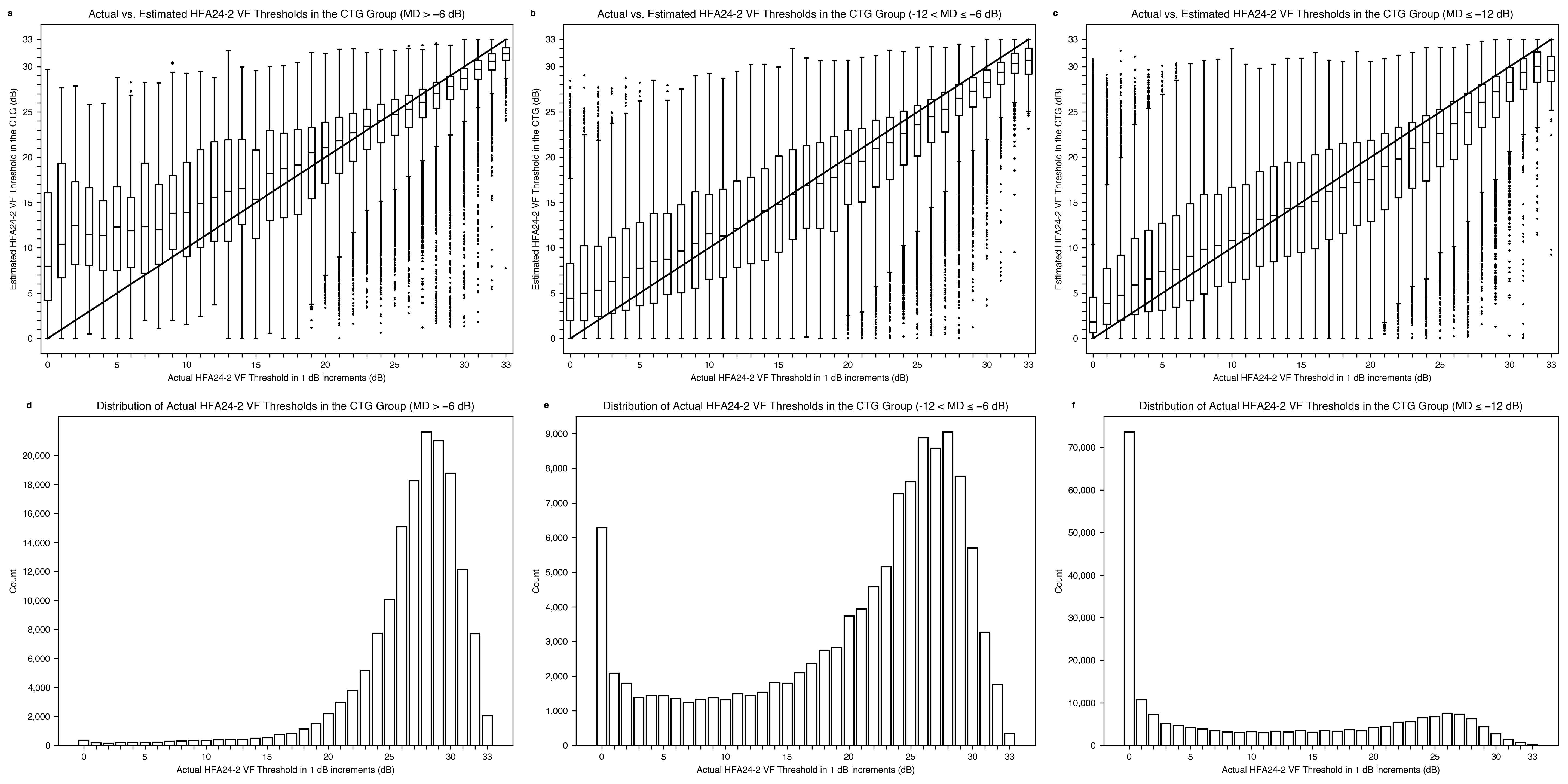

Supplement: Supplementary file 4 — Supplementary Material 4 [file 41598_2025_98511_MOESM4_ESM.tiff]
