# Supplementary material for: Automated learning of glaucomatous visual fields from OCT images using a comprehensive, segmentation-free 3D convolutional neural network model
Source: Sci Rep. 2025 Apr 18;15:13395. doi: 10.1038/s41598-025-98511-0 (PMC12008402; doi:10.1038/s41598-025-98511-0)
Supplement: Supplementary file 5 — Supplementary Material 5 [file 41598_2025_98511_MOESM5_ESM.docx]

**Supplementary Figure 1.** Boxplots showing the relationship between OCT focus (x-axis) and MAE (y-axis) in the CTG for HFA24-2 versus HFA10-2, and Threshold versus MD.

- **(a)** HFA24-2 Threshold pointwise MAE.
- **(b)** HFA10-2 Threshold pointwise MAE.
- **(c)** HFA24-2 MD MAE.
- **(d)** HFA10-2 MD MAE.

The polynomial slopes (in dB per diopter) were:
- (a): -0.0030 dB/D
- (b): -0.079 dB/D
- (c): +0.046 dB/D
- (d): -0.066 dB/D

A linear regression (solid black line) is overlaid on each boxplot. The near-zero slopes in all panels indicate that refractive error (myopia or hyperopia) has minimal impact on the model’s performance. Specifically, the polynomial slopes and y-intercepts suggest that MAE remains relatively stable across a wide range of OCT focus values.

OCT: optical coherence tomography, MAE: mean absolute error, CTG: Comprehensive Training Group, HFA: Humphrey Field Analyzer, MD: mean deviation (dB),

**Supplementary Figure 2.** Comparison of actual vs. estimated HFA24-2 VF thresholds and their distributions for mild, moderate, and severe glaucoma subgroups as defined by MD values in the CTG.

- **(a), (b), (c)**: Boxplots of actual (x-axis) vs. estimated (y-axis) HFA24-2 VF thresholds for three MD-defined groups, respectively:
  (a) **mild** (MD > −6 dB, n =3,034 pairs),
  (b) **moderate** (−12 dB < MD ≤ −6 dB, n = 2,246 pairs),
  (c) **severe** (MD ≤ −12 dB, n = 4,134 pairs).
  Pearson’s r (Spearman’s ρ) for each group was as follows:
  - mild: r = 0.711 (ρ = 0.734),
  - moderate: r = 0.800 (ρ = 0.800),
  - severe: r = 0.821 (ρ = 0.792),
  all p-values = 0.
- **(d), (e), (f)**: Distributions (y-axis = count) of Actual HFA24-2 VF thresholds (x-axis) for the same mild (a), moderate (b), and severe (c) subsets.

To avoid unrealistically high sensitivity outliers, any measured threshold exceeding the age-matched normal value at that test location was reassigned to the corresponding normal threshold.

While the model shows good overall performance across severity groups, accuracy varies according to defect patterns. Notably, in mild cases (MD > −6 dB), the model shows decreased accuracy when estimating isolated deep defects. As shown in the distribution plot (d), the number of test points with low sensitivity values within the mild group is relatively small compared to the moderate and severe groups. This limited sample size of localized deep defects in otherwise healthy visual fields may contribute to the model's reduced performance in accurately predicting these specific patterns. This finding suggests that the model may rely more on patterns of global loss when estimating visual field sensitivities, which has implications for its clinical application in early glaucoma detection where focal defects often precede diffuse loss.

HFA, Humphrey Field Analyzer; VF, visual field; MD, mean deviation; CTG, Comprehensive Training Group.

**Supplementary Figure 3.** Comparison of measured versus estimated HFA24-2 VF thresholds and their absolute errors in the central versus peripheral regions, defined by whether test points lie within or beyond 15° eccentricity.

- **(a) and (b)** Boxplots of actual (x-axis) vs. estimated (y-axis) HFA24-2 VF thresholds in the CTG. Panel (a) shows data for the central 15° (34 test points in a 6×6 grid excluding the two points corresponding to the Marriott blind spot), while panel (b) shows data for the peripheral region (>15°, 18 test points outside the 6×6 grid). In the central region, Pearson’s r = 0.883 and Spearman’s ρ = 0.878 (both p = 0); in the peripheral region, r = 0.867 and ρ = 0.838 (both p = 0).
- **(c) and (d)** Boxplots of the absolute error (y-axis) vs. Actual HFA24-2 VF thresholds (x-axis) for the same central (c) and peripheral (d) subsets in the CTG.

For any measured threshold that exceeded the age-matched normal value at the same test location, the label was reassigned to that normal threshold to avoid unrealistically high sensitivity outliers, particularly in peripheral measurements. Although the peripheral region (>15°) extends beyond the OCT scan coverage, our model still achieves reasonable estimation. A slight increase in error was observed at the higher-threshold range (~32 dB) in the peripheral area.

HFA, Humphrey Field Analyzer; VF, visual field; CTG, Comprehensive Training Group; OCT, optical coherence tomography.

**Supplementary Figure 4.** Pointwise comparisons of actual (x-axis) versus estimated (y-axis) HFA24-2 VF thresholds for 52 test points (excluding the 2 points corresponding to the Marriott blind spot) in the CTG. Boxplots display the distribution of estimated thresholds (y-axis) for each actual threshold (x-axis) at each test point, with colored borders grouping test locations according to the 10 clusters used in the Glaucoma Hemifield Test. Data from left eyes are horizontally flipped and merged in a right-eye orientation. For any measured threshold exceeding the age-matched normal value at a given test location, the label was reassigned to that normal threshold to avoid unrealistically high sensitivity outliers. The figure shows that estimation accuracy correlates with the length of visible nerve fiber layer trajectory captured within the OCT scan area. Specifically, nasal visual field points (corresponding to temporal retinal areas well-covered by the OCT scan), colored in red (superonasal cluster) and blue (inferonasal cluster), show better estimation performance than temporal visual field points (corresponding to nasal retinal areas partially outside the scan boundaries). Estimation errors notably increase for the temporal VF points (located to the right of the Marriott blind spot) in severe cases, as these regions correspond to retinal areas that fall outside the OCT scan's 9 × 9 mm coverage.

HFA, Humphrey Field Analyzer; VF, visual field; CTG, Comprehensive Training Group; OCT, optical coherence tomography.
